# Orthopedic Devices for Skeletal Class III Malocclusion Treatment in Growing Patients: A Comparative Effectiveness Systematic Review

**DOI:** 10.3390/jcm13237141

**Published:** 2024-11-25

**Authors:** Angelo Michele Inchingolo, Alessio Danilo Inchingolo, Irma Trilli, Laura Ferrante, Angela Di Noia, Elisabetta de Ruvo, Andrea Palermo, Francesco Inchingolo, Gianna Dipalma

**Affiliations:** 1 Department of Interdisciplinary Medicine, University of Bari “Aldo Moro”, 70124 Bari, Italy; angeloinchingolo@gmail.com (A.M.I.); ad.inchingolo@libero.it (A.D.I.); trilliirma@gmail.com (I.T.); lauraferrante79@virgilio.it (L.F.); angeladinoia@libero.it (A.D.N.); studio.deruvo@libero.it (E.d.R.); giannadipalma@tiscali.it (G.D.); 2 College of Medicine and Dentistry Birmingham, University of Birmingham, Birmingham B46BN, UK; andrea.palermo@2004libero.it

**Keywords:** skeletal class III malocclusion, orthopedic treatment, orthopedic and functional devices, maxillar retrusion and underdevelopment, jaw protrusion and hyper development

## Abstract

**Background/Objectives:** Skeletal Class III malocclusion (Cl III) presents a significant orthodontic challenge, particularly in growing patients, requiring interceptive treatment to achieve effective functional and aesthetic correction. This review aims to compare various orthopedic devices and therapeutic protocols used in Cl III correction, identifying the most effective options in an interceptive context. **Methods:** We searched the PubMed, Scopus, and Web of Science databases for studies published between 1 January 2003, and 19 July 2023. Inclusion criteria included English language, human studies, open access, and studies addressing Cl III correction with interceptive orthopedic devices. **Results:** Exclusions included in vitro, animal, off-topic studies, reviews, meta-analyses, and articles in languages other than English. After removing duplicates, 30 articles were selected from a total of 1193 results. **Conclusions:** The application of orthopedic devices in growing patients can lead to rapid improvement of Cl III malocclusion, although each device has specific effects on the surrounding skeletal structure.

## 1. Introduction

A change in the sagittal connection between the maxilla and mandible with mandibular protrusion, and/or hypermandibulia, accompanied or not by retrusion, and/or underdevelopment, of the upper jaw is referred to as skeletal class III malocclusion (Cl III) [1,2]. In the context of horizontal cephalometric analysis, key measurements include SNA, SNB, and ANB angles, which help assess the anteroposterior relationship between the maxilla and mandible. An ANB value of 2 ± 2 degrees typically indicates a Class I skeletal pattern, with deviations suggesting Class II (>4 degrees) or Class III (<0 degrees) patterns [3]. These measurements help differentiate whether discrepancies in jaw relationships are due to excess or deficiency in the maxilla or mandible [4,5]. Additionally, the Wits appraisal offers further insight into the anteroposterior jaw relationship and skeletal divergence [6]. Normal Wits values range from −1 mm ± 2 mm. A negative Wits value indicates increased divergence as the occlusal plane steepens [7,8]. From a dental perspective, Class III malocclusion, as defined by Angle in 1907, involves a deviation in dental occlusion where the mesiobuccal cusp of the upper first molar contacts the distobuccal cusp of the lower first molar, potentially resulting in an anterior crossbite with reversed tooth relationships [9,10].

**Figure 1:**
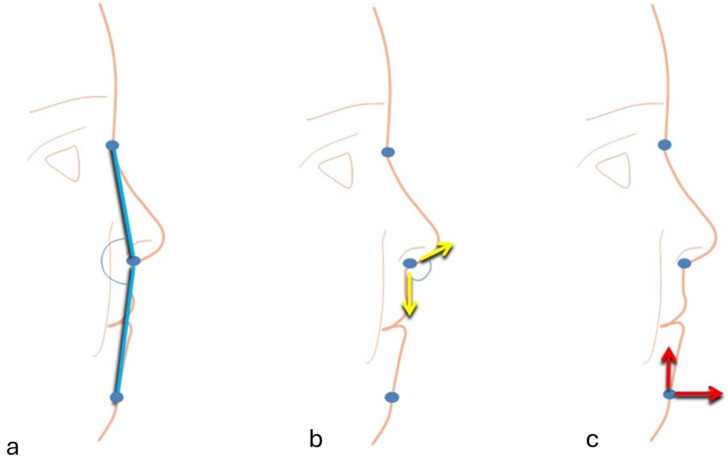
Profile characteristics in a Cl III: (**a**) concave profile and reduced middle facial third, (**b**) wide nasolabial angle, (**c**) pronounced chin.

As a physiological attempt to repair the inverted bite, the discord of the skeletal bases is occasionally accompanied by dental compensation, with maxillary dento-alveolar protrusion and mandibular dento-alveolar retrusion: a frontal or lateral crossbite [11,12]. The low posture of the tongue, which may or may not be accompanied by macroglossia, and the concave profile, which is characterized by a real or apparent deficit of the middle third of the face, a wide nasolabial angle, a prominent lower lip with more exposure of the lower incisors, and a prominent chin (Figure 1), are additional pathognomonic features that may be present in this dysgnathias [8,13,14].

Cl III is a rare malocclusion in the Caucasian population, with an incidence of 5% in Italians, while it is predominant in Asian ethnicities, with incidence rates up to 14% in Chinese and Japanese populations and 3.4% in Indian populations.

The patient’s genetic background also affects how bad this illness is. Although this malocclusion is easy to spot, it is incredibly challenging to fix. Cl III disharmony is one of the most challenging and surprising orthodontic malocclusions to treat. It is usually possible to prevent catastrophic future malocclusions, frequently of the skeletal type, by determining the appropriate timing of treatment in the face of orthodontic disorders deemed “urgent,” such Cl III [15,16].

Early correction of malocclusion favors dentofacial development and may help prevent the growth of more severe malocclusions in late adolescence [17,18].

Throughout the years of development, numerous interceptive treatment approaches, such as permanent appliances, functional portable appliances, and skeletal anchorage systems, have been presented to address this malocclusion [19,20,21].

**Figure 2:**
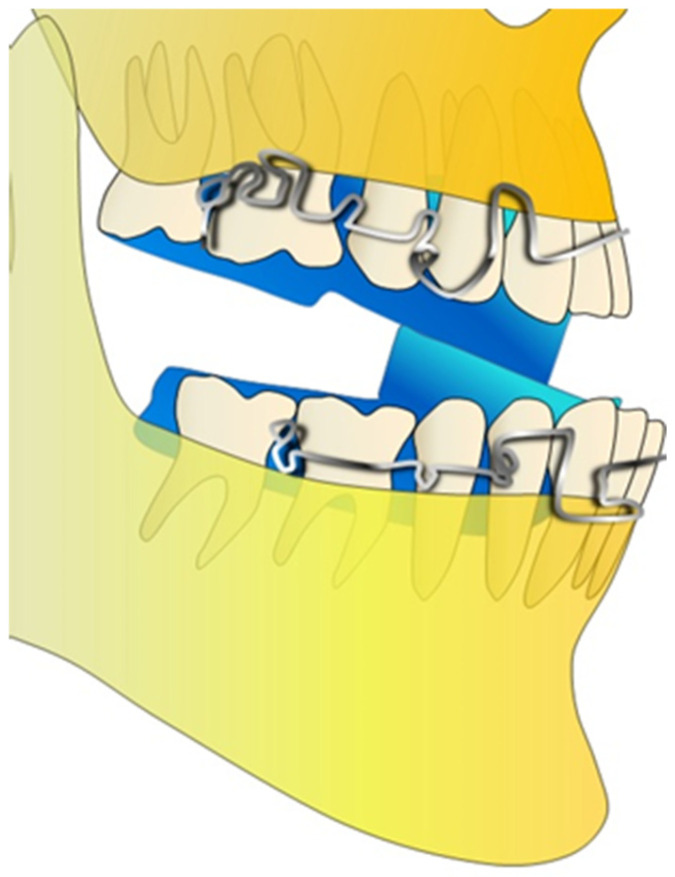
Design of the double-plate appliance.

One of the most typical issues with extraoral therapy is low patient compliance. Frankel III, Bionator III, and double-plate appliances (DPAs) are examples of functional intraoral equipment. Even though some studies claim that it had a stronger dental and less skeletal effect than facemask (FM) therapy, the latter, which was built with reverse resin planes, has produced excellent outcomes in maxillary protrusion. However, prior research showed that Cl III was effectively treated with good vertical dimension control when DPAs and FMs were combined (DPA-FM). The DPAs can have two Cl III elastics. The appliances had lower labial bows with hooks for Cl III elastics in the anterior area and modified Adams clasps at the molar region. (Figure 2) [22].

Functional devices, such as the Bionator or removable mandibular retractor (RMR), Frankel III appliance, reverse twin block (RTB), and orthopedic appliances, including FMs and the chin rest, have shown excellent results in correcting III dysgnathia class [23,24,25].

However, FMs indicated stronger skeletal effects in maxillary protrusion, while RTB primarily revealed dentoalveolar alterations [26,27,28,29,30,31]. In any event, early therapy with functional maxillary protrusion equipment has shown to be more successful than using orthopedic devices to inhibit mandibular growth, such as the chin rest, which has had mixed results [32,33,34]. 

Nowadays, the combination of facial masks (FMs) and rapid palate expansion (RME) is the recommended treatment approach for correcting class III malocclusion in teenagers. Transverse palate expansion does not by itself result in increased maxillary protrusion, even though RME offers several benefits. Rather, the profile and Class III malocclusion are improved by the therapeutic effect of FMs, which results in posterior rotation of the jaw and maxillary protrusion [35,36,37,38,39,40]. To further encourage maxillary protrusion and enhance therapy results in Cl III patients, a novel method known as alternate rapid palate expansion and contraction (Alt-RAMEC) makes use of springs. Research has verified that the Alt-RAMEC approach in conjunction with FMs yields protrusion outcomes that are comparable to those of the conventional RME/FM protocol [41,42,43,44]. In situations when there is a maxillary deficiency, a FM for protrusion is advised. Unwanted side effects, including elevated facial height, excessive upper incisor proclivation, and anterior displacement of upper molars, have been documented in trials that combine RME with dental anchoring. For Cl III patients with hyperdivergent growth patterns, the Hybrid Hyrax device with skeletal anchoring was created as an efficient substitute to lessen these consequences.

**Figure 3:**
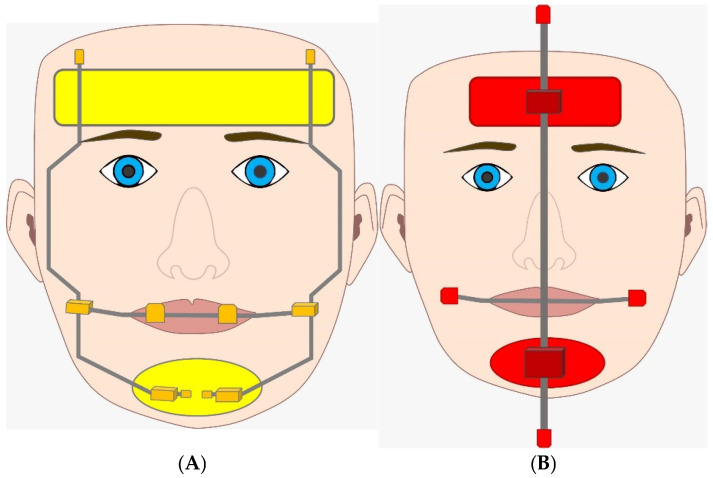
(**A**) Delaire mask device as a treatment of Cl III. (**B**) Petit mask device as a treatment of Cl III.

Since FM presses the mandible forward and applies indirect stresses to the still-active peri-maxillary sutures, it has been shown that early treatment of Cl III with FM and RME can successfully rectify malocclusion by promoting bone apposition in the sutural areas [19,45]. The combination of RME and FM provides more effective maxillary protrusion. With traditional FM therapy, such as Delaire or Petit devices, the mandible rotates backward, while upper and lower incisors tilt toward labial and lingual, respectively [46]. This phenomenon results in increased lower facial height and reduced overbite. However, it has been observed that FMs with dental projection can generate side effects such as extrusion of maxillary molars and excessive proclivation of the incisors, which are problematic when dental arch maintenance is needed [47,48] (Figure 3).

The Hybrid Hyrax RME appliance was developed in an effort to create an absolute anchorage system for maxillary protrusion utilizing bone-anchored devices. It is a dental and skeletally anchored device that places two mini-implants in the palate. Rubber bands are used to keep the Hybrid Hyrax Expansion Appliance hooks in place as the patient receives FM therapy after expansion. RPE Hybrid Hyrax, a bone-anchored prosthetic, lessened the adverse dental consequences of RME appliances [49]. FM and reverse twin-block (RTB) therapy has been shown to be very effective in the early treatment of Cl III [50].

Dental impacts are the predominant side effects of the RTB device, with little skeletal effects, and are characterized by proclination of the upper incisor and retroclination of the lower incisor [51]. FMs combined with jaw expanders have been shown to be more effective in the early than late stages of skeletal maturation [4]. Depending on how the treatment is going, therapy is frequently not limited to just one type of equipment but instead might incorporate a variety of appliances [30,52]. According to a 2016 study by Jamilian A. et al., a Cl III in a growing patient may soon improve with the use of orthopedic devices, but each device has a different effect on the nearby skeletal model. For this reason, the goal of our investigation is to look into the most significant studies from a scientific standpoint that examine the correction of class III skeletal malocclusion, comparing the various functional and orthopedic devices and the various treatment approaches [53].

One of the most common devices for the treatment of maxillary deficit is the inverted chin cap. Studies compared the effects of the face mask and reverse chin rest on growing patients with upper jaw deficit, typical of skeletal class III. Cephalometric results showed similar upper jaw advancement in both appliances, with increased SNA angle and decreased lower mandibular incisor inclination, with no significant differences between the two approaches. Thus, it can be inferred that the reverse chin rest also provides effective results in promoting forward movement of the upper jaw in growing patients [54].

Currently, options for expanding the maxilla in patients with maxillary deficit include a lingual plate. This approach has been shown to be effective in treating crossbite and lateral maxillary contraction, providing improved transverse palatal width and occlusal stability over time. In particular, the use of a removable palatal plate in mixed dentition is a common and established option for the management of such maxillary deficits. A recent study also explored the use of the double lingual plate combined with the face mask for the correction of class III malocclusion, showing that this configuration helps to control mandibular incisor movement, promoting maxillary advancement without the risk of tooth retraction. The results showed significant changes in molar relation and overjet, positioning the lingual plate among the effective approaches for maxillary deficit in growing patients [55].

## 2. Materials and Methods

### 2.1. Protocol and Registration

This study was conducted according to the standards of Preferred Reporting Items for Systematic Reviews and Meta-Analyses Extension for Scoping Reviews (PRISMA-ScR) [56], and it was registered at PROSPERO under the ID 451982.

### 2.2. Search Processing

A search on PubMed, Scopus, and Web of Science was performed (Table 1) to find papers that matched the topic of the treatment of Cl III with interceptive orthopedic devices in comparison, dating from 1 January 2003 to 19 July 2023. The search strategy used the following Boolean keywords:

Cl III AND (orthopedic treatment OR orthopedic devices OR FM OR Delaire).

### 2.3. Inclusion Criteria

The following inclusion criteria were considered: (1) studies that investigated the treatment of Cl III with interceptive orthopedic devices in comparison, (2) randomized clinical trials, retrospective and observational studies with patient ages ranging from 5 to 14 years old, (3) English language, (4) full–text available, (5) open access studies.

Papers that did not match the above criteria were excluded.

The review was conducted using the following PCC criteria:

Population: growing patients with an age range from 5 to 14 years old, male and female, with Cl III;

Concept: treatment of Cl III with interceptive orthopedic devices in comparison;

Context: public health care.

### 2.4. Exclusion Criteria

The exclusion criteria were as follow: (1) animal studies; (2) in vitro studies; (3) off-topic; (4) case report, case series reviews, letters, or comments to editors; (5) no English language.

### 2.5. Data Processing

Five reviewers (A.D.N., L.F., I.T., and E.D.R.) independently consulted the databases to collect the studies and rated their quality, based on selection criteria. The selected articles were downloaded into Zotero (version 6.0.15). A meeting with a senior reviewer resolved any disagreements between the reviewers (F.I.).

### 2.6. Quality Assessment

The quality of the included papers was assessed by two reviewers, RF and EI, using the ROBINS-I, a tool developed to assess risk of bias in the results of non-randomized studies that compare health effects of two or more interventions. Seven points were evaluated, and each was assigned a degree of bias. A third reviewer (FI) was consulted in the event of a disagreement until an agreement was reached.

## 3. Results

### 3.1. Study Selection and Characteristics

**Figure 4:**
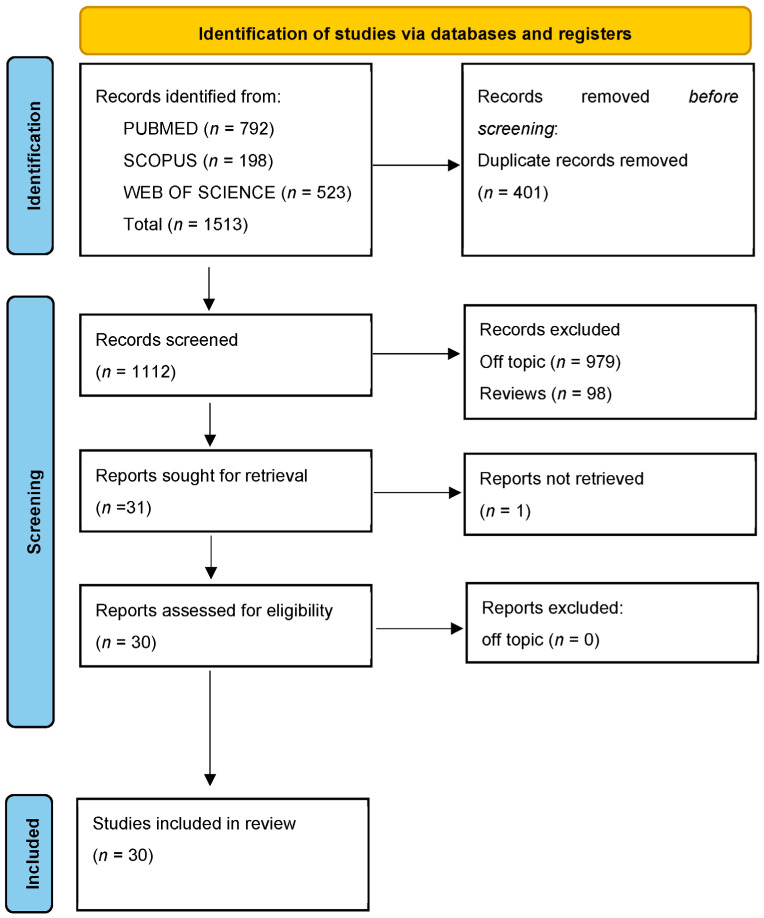
Indicators from the database search and the PRISMA (Preferred Reporting Items for Systematic Reviews and Meta-Analyses) flowchart illustrating the literature search process.

No papers were found during the manual search; however, the electronic database search yielded a total of 1513 articles (Scopus: 198, PubMed: 792, and Web of Science: 523). Following the removal of duplicates, 1112 studies underwent screening based on title and abstract content, with a specific focus on the treatment of Cl III using interceptive orthopedic devices for comparison. Out of these, 1081 articles did not meet the inclusion criteria (979 were off-topic, and 98 were reviews), and one report was not retrieved, resulting in the selection of 30 records. After assessing eligibility, these 30 records were chosen for qualitative analysis. The selection process and a summary of the selected records can be found in Figure 4 and Table 2, respectively.

### 3.2. Quality Assessment and Risk of Bias

**Figure 5:**
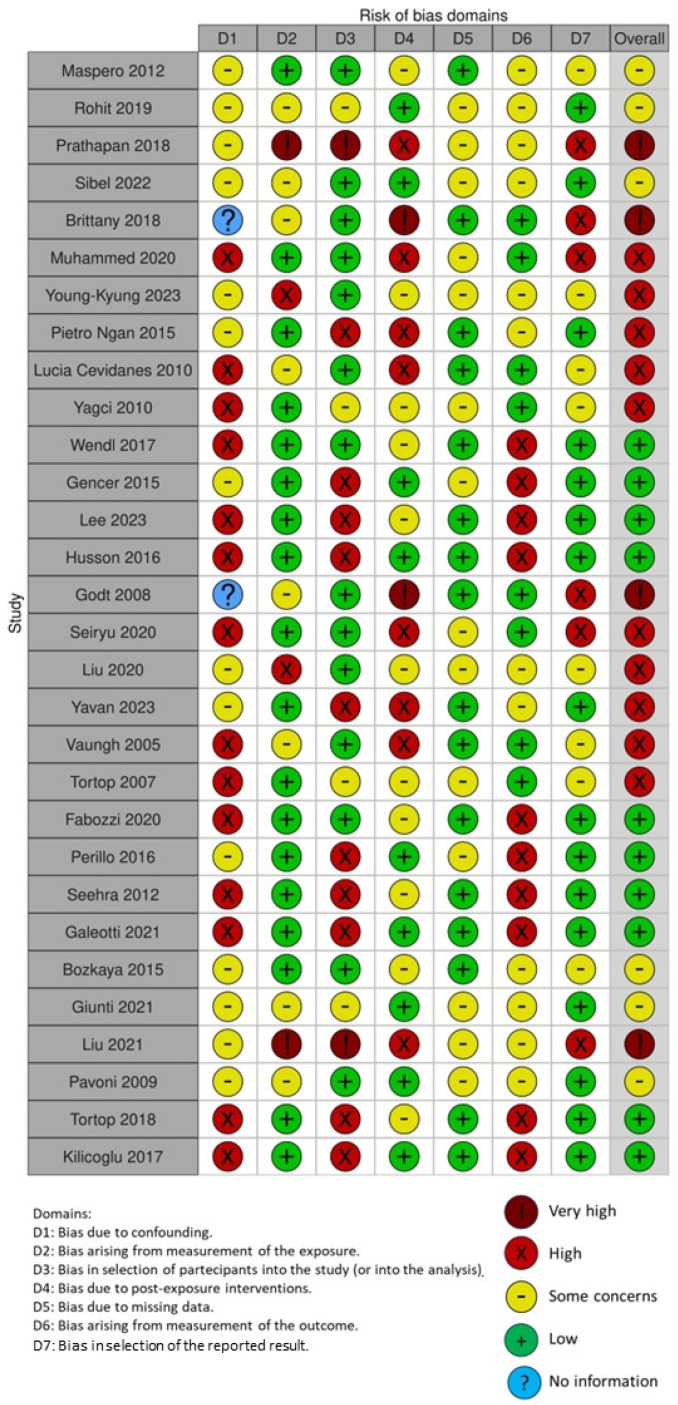
Risk of bias assessment using ROBINS-I tool [17,18,22,30,35,36,37,44,49,51,57,58,59,60,61,62,63,64,65,66,67,68,69,70,71,72,73,74,75,76].

The risk of bias in the included studies is reported in Figure 5. Regarding the bias due to confounding, most studies have a high risk. The bias arising from measurement is a parameter with low risk of bias. Most studies have low risk of bias due to bias in selection of participants. Bias due to post-exposure cannot be calculated due to high heterogeneity. The bias due to missing data is low in most studies. Bias arising from measurement of the outcome is low. Bias in the selection of the reported results is high in many studies. The results show that 10 studies have a low risk of bias, 10 studies have a high risk of bias, 3 have a very high risk of bias, and the remainder have a questionable risk of bias.

## 4. Discussion

Groups of patients with different orthopedic devices were compared, and different therapies in the treatment of Cl III were analyzed.

In a comparative analysis of orthopedic devices for treating Class III malocclusion, various studies revealed significant improvements in patient outcomes. For instance, Maspero et al. found that the transverse sagittal maxillary expander (TSME) led to greater SNA angle increases and maxillary dentoalveolar advancements compared to the rapid Hyrax expander (RME/FM) [22]. In a separate study by Rohit et al., among patients aged 6 to 12, RTBLP-RME showed superior sagittal improvements and less incisor proclination than FM-RME, highlighting the efficacy of targeted treatments based on individual patient characteristics [35].

A series of studies have examined various treatment protocols for correcting SCIIIM in growing patients. Prathapan Parayaruthottam et al. compared the Alt-RAMEC and RME protocols in 18 patients, finding both effective in correcting bite relationships [68]. The Alt-RAMEC protocol resulted in maxillary proclination and significant soft tissue improvement. Further analyses by Akbulut et al. [17] and Fisher et al. [44] assessed Alt-RAMEC/FM and RME/FM protocols, with both showing positive orthopedic changes but the Alt-RAMEC/FM group exhibiting better SNA, ANB, and overjet improvements. Muhammed Hilmi Buyukcavus et al. evaluated RME, modified Alt-RAMEC, and skeletal anchorage, finding the latter to be most effective [57], while Youn-Kyung Choi et al. reported that screw anchorage yielded greater maxillary advancement and fewer side effects compared to conventional methods. Overall, the studies highlight the benefits of specific protocols tailored to patient needs, particularly those utilizing skeletal anchorage for optimal outcomes [58].

A few years earlier, a study by Pietro Ngan et al. sought to compare the differences in results between the Hybrid Hyrax expansion appliance with bone support and protraction template (20 patients) and RPE appliance with dental support and protraction template (20 patients). For comparison, a control group of 20 Cl III patients who were not receiving treatment was used. A dental-based RPE was built by attaching bands to the back teeth and turning the expansion screw twice a day for one to two weeks. On the other hand, bands were placed on the first permanent molars, and anchorage was provided by micro-implants in the bone-supported hybrid RPE. The protraction template was one piece, with hooks for rubber bands and an anterior wire that could be adjusted. In both treatment groups, the mandible moved backward, and the maxilla moved forward, according to cephalometric analysis, which helped to increase Wits and ANB ratings [77].

Both treatment groups saw a significant improvement in the overjet and molar relation; however, the RPE group with bone anchorage had more downward movement of the maxillary incisors, which helped to maintain the overbite, whereas the group with dental support had more forward movement of the maxillary incisors, which contributed to a greater increase in overjet than the group with bone anchorage.

In comparison to the group receiving dental anchorage, the latter also displayed less maxillary retraction and less mandibular rotation in a clockwise direction [78].

This skeletal-anchored supported mini-screw is a promising alternative treatment for the repair of Cl III individuals with a hyperdivergent development pattern since it minimizes dental adverse effects as a result of the insertion of two mini-implants to the Hybrid Hyrax RPE [18].

In a study by Lucia Cevidanes et al., two Class III correction protocols—Bone Anchored Maxillary Protraction (BAMP) and Rapid Maxillary Expansion with a face mask (RME/FM)—were compared in terms of dentoskeletal changes in patients. Results indicated that BAMP achieved superior maxillary advancement and midface lengthening, with greater vertical control than RME/FM, avoiding clockwise mandibular rotation. While RME/FM resulted in increased facial height and lower incisor inclination, BAMP reduced maxillomandibular divergence, showing more favorable control over mandibular rotation and vertical growth adjustments [79].

The BAMP group improved the molar connection substantially more than the RME/FM group did. The BAMP group also reduced negative dental alterations, including a lingual inclination of the lower incisors, which helped to lessen the difference in overjet change between the two groups [49].

The analysis of the studies confirms that all treatment methods are effective in addressing Class III with maxillary growth deficiencies. However, treatment plans should be tailored based on the patient’s unique diagnostic profile, age, growth stage, treatment goals, and budget. Favorable outcomes, such as maxillary advancement and improved sagittal alignment, were noted across groups, with both modified SEC III and RME/FM showing similar effects. One difference emerged in palatal plane tilt: RME/FM tilted it upward, while SEC III showed a slight downward tilt [59,70].

RTB appliance effects do not produce effects on dentition but rotational effects on the tooth bases. PFM therapy is skeletal in nature, with the greatest impact on maxillary position, sagittal advancement, and an average increase in SNA of +2.1. Minimal effect on SNA (mean, 1.2°) and increased reflex overjet is obtained with PFM use, with a greater skeletal contribution than dental contribution with protraction headgear therapy [51,80].

The mandibular position assessed by SNPg and ANPg angles showed a statistically significant decrease with the RME/FM protocol compared with the PS3 group. The CoGoMe angle showed a significant decrease in the RME/FM group compared with the PS3 group. Force applied to the chin in the cranial and posterior directions redirected mandibular rotation. The PS3 group showed good control of mandibular divergence with respect to the anterior skull base and a decrease in mandibular clockwise rotation. The use of PS3 is recommended in hyperdivergent patients. The PS3 group shows greater protrusion of upper incisors and retrusion of lower incisors than the RME/FM group. This might depend on the fact that PS3 covered the upper and lower incisors, whose inclinations were affected by sagittal forces [60].

The success of Cl III treatment with DPA or DPA-FM appliances have been reported, but no comparative studies have been performed in the literature. In this study, significant increases in SNA angle and CoA length showed that maxillary growth was the main effect of the therapies.

In both DPA and DPA-FM groups, a lip version of the upper incisor and tilting of the lower incisors occurred [36].

RME/FM therapy produced significantly greater clockwise mandibular rotation (SN-Mand. Pl. +1.8 degrees), associated with significantly greater increases in intermaxillary divergence (Pal. Pl. -Mand. Pl. +2.5 degrees) compared to RMR, which showed 1.2 mm and 1.4 mm maxillary advancement of Point A at Nasion, respectively [62].

Alt-RAMEC followed by FMs was compared. However, limited studies have reported the simultaneous use of Alt-RAMEC with FMs. Significant differences were shown between the groups in treatment duration, which was reduced by about 20% in the Alt-RAMEC/FM group (6.04 months) compared with the FM group (7.99 months) and the RME/FM group (7.35 months). Several studies have reported periodontal damage from tooth-worn devices compared with the protocol on the bone. Because orthopedic force is transferred through skeletal anchorage, the risk of periodontal damage could be minimized with mini-implants anchored in the palate [67].

Craniofacial response to orthopedic treatment with FM/BB and RME/FM protocols consisted of skeletal dental modifications in both sagittal and vertical planes. This comparison of the two treatments for prepubertal orthopedics revealed that FM/BB and RME/FM showed no significant differences in short-term skeletal, dental, and soft tissue effects [69].

The comparison between FM and MTTBA was evaluated on cephalograms to assess the posterior mandibular space.

Both treatment groups showed the lower incisors’ tongue-version (L1/NB). The lower incisor in Cl III develops a tongue-like form because of FMs and some detachable appliances, which helps to rectify the malocclusion. Significant increases in branch width were seen in all groups [73].

The modified Jasper Jumper device rotates the lower dentition counterclockwise and exerts a downward and backward tension on the lower molars. Movement is produced in the region of the backward molars, together with distal tilting and intrusion of the lower molars and a reduction in the tilt of the occlusal plane. Backward molar movement, distal tilting and incursion of the lower molars, and a decline in the slope of the occlusal plane are all caused by this. The lower molar vertical reference line’s (mi/OLp) distance was assessed in our study for both groups, and no discernible difference in this parameter was found. As a result of the difference between the is/Olp and ii/Olp distances, Pancherz noticed a change in the overjet [64].

Yagci et al. [37] compared the conventional FM and the modified FM with rapid maxillary expansion (RME). In a group of 24 patients, strong elastics (500 g) and a bonded full coverage maxillary acrylic splint expander with vestibular hooks were used. The modified FM was used by a second group of 24 patients, who also wore a modified bonded rapid maxillary expansion appliance with full occlusal coverage, a facebow that was created especially for them, an FM, and heavy elastics (500 g). Intra-group variations were examined using the non-parametric Wilcoxon’s test. While with the Kruskal–Wallis test, the inter-group comparison was evaluated. The statistical analysis was carried on with the Mann–Whitney test and with Bonferroni’s correction. Yagci et al. demonstrate how FM treatments with expansion caused the mandible to shift backward and downward in both treatment groups, whereas the maxilla advanced anteriorly and translated without rotating. Also, Vaughn et al. [74] compared the application of FMs with or without expansion. Patients were randomly divided into a group who received the FM with palatal expansion, a second group with the FM without palatal expansion, and a control group. Statistical analysis was executed with Student’s *t*-test and showed no significative difference between the two groups with FMs with or without expansion, while significative changes were observed between these groups and the control sample, demonstrating that the early therapy with these appliances with or without expansion can be effective. Tortop et al. [72] exhibited a maxillary forward displacement in both the groups, and the single FM and the group with FM exhibited much more palatal extension than the control group. The FM therapy with or without expansion can be efficacious for Cl III treatment, giving dental and skeletal achievements [81,82,83].

Wendl et al. [75] studied the use of a chin cup or FM for Cl III correction in growing patients, studying in a retrospective way cephalograms, casts, and orthopantomograms of patients. These data were analyzed at T0, after the treatment (T1), and after 15–20 years (T2) to evaluate the long-term stability of the treatment. The effectiveness of both appliances in the correction of third class had no discernible differences. A significant difference was found for what concerns the long-term stability and the risk of recurrence; the chin cup has a lower long-term continuity due to immoderate mandibular growth or due to the loss of maxillary catch-up growth.

Gencer et al. [61] analyzed two types of therapy with FMs. In a subgroup of 15 patients, the classic FM was used for the treatment of Cl III. In another subgroup of 15 patients, it was applied as a double-plate appliance/FM (DPA-FM) combined therapy. Finally, it was utilized including a 15-patient control group. Also, in this study, no differences were individuated for the effectiveness of these two appliances, but significant changes were seen in this case for sagittal modifiers. Probably because of the acrylic blocks of the double-plate appliances, which have a restrictive power on the dental arch, in the subgroup that used the classic FM, a significant sagittal difference in the inferior incisors and pogonion was noticed that is absent for the other group.

Lee et al. [65] compared two types of face masks (PFFS and PTF) for Class III treatment, finding no significant differences except in overbite. Husson et al. [63] assessed FMs and rapid palatal expansion (RPE), noting both were effective for Class III, but the modified tandem appliance (MTA) outperformed the FM in controlling jaw rotation. Godt et al. [30] reported increases in overjet and changes in mandibular angles, supporting early orthopedic therapy. Seiryu et al. [71] found FMs combined with mini-screws led to better skeletal outcomes with fewer side effects. Liu et al. [66] highlighted faster results with modified appliances compared to banded ones, and Yavan et al. noted both RF and FM/RPE improved alignment, with RF showing less skeletal impact [78,84].

In the limitations of the studies analyzed, the difficulty of evaluating repeatable results due to the different and non-constant collaboration of the growing patient should be considered first. Poor cooperation is one of the most prevalent issues for functional interceptive therapy [85,86]. There is a lack of sufficient studies in the literature comparing the outcomes of the various devices in the treatment of Cl III [87]. There is a need for further studies, and with a larger patient sample, to evaluate the stability of the various long-term therapeutic protocols. The lack of untreated mild Cl III patients as controls is a major limitation of the review. It would be unethical for control patients to go untreated and expose control subjects to radiation despite the need for immediate intervention. However, this source of bias is very unlikely to have affected the results. Furthermore, only conventional 2D cephalometric studies are available in the literature; these further studies should exploit three-dimensional images via CBCT (Cone Beam Computed Tomography), which has several advantages over lateral cephalometries, to evaluate changes in skeletal and dentoalveolar regions in a more precise and detailed way, offering clinicians greater awareness in operational treatment choices [88,89,90]. This review confirms that traditional devices like the rapid palate expander (RME) and face mask (FM) remain effective for Class III treatment. However, innovative methods using skeletal anchors show significant benefits, including enhanced maxillary advancement and reduced dental side effects. The findings emphasize the importance of tailored device combinations based on individual patient needs, suggesting that a flexible and personalized approach may yield better results than standardized protocols.

## 5. Conclusions

Devices and treatment protocols for Class III dentoskeletal correction in growing patients produce significant skeletal and dental improvements, especially when treatment is initiated early. Combined therapies of orthopedic and functional devices are more effective in correcting growth than the exclusive use of functional devices, helping to counteract any adverse dental effects. Despite the great results of skeletal anchors, there is no evidence that skeletal anchoring provides greater short-term treatment effects, nor does it result in greater long-term stability than other treatment approaches for Class III malocclusions. Some limitations lie in the two-dimensional nature of cephalometric analyses and the lack of control groups for ethical reasons. Future studies with larger samples and three-dimensional imaging could improve outcome monitoring and optimize treatment choices, confirming the value of an individualized approach according to patient characteristics.

## Figures and Tables

**Table 1:** Database search indicators.

Articles screening strategy	KEYWORDS: A: Class III malocclusion; B: orthopedic treatment; C: orthopedic devices; D: FM; E: Delaire
	Boolean Indicators: (A) AND (B OR C OR D OR E)
	Timespan: from 1 January 2003 to 19 July 2023
	Electronic databases: PubMed; Scopus; WOS

**Table 2:** Descriptive summary of item selection.

Authors (Year)	Study Design	Patient’s Number/Gender/Mean Age	Appliances	Analyzed Parameter	Materials and Methods	Outcomes
Akbulut S. et al. (2022) [17]	Comparative study	30 pz Grp. 1: 10 M, 5 F; mean age 10.99 ± 1.80 yo. RME/FM Grp 2: 10 M, 5 F; mean age 11.61 ± 1.20 yo.	Alt-RAMEC/FM vs. RME/FM	Lateral cephalometric radiographs: skeletal, dental, and soft tissue changes were analyzed.	30 patients who received FM therapy after Alt-RAMEC or RME protocols. The Alt-RAMEC group activated expansion screws for one week, while the RME/FM group performed screw activation based on patients’ needs.	The maxilla significantly advanced sagittally in both groups. In contrast to the RME/FM group, the Alt-RAMEC/FM group showed statistically better gains.
Bozkaya et al. (2015) [36]	Retrospective study	40 patients (13 with DPA, 15 patients with DPA-FM, and 12 without treatment)	DPA versus DPA-FM	Increased SNA and ANB angles in groups with DPA_FM compared with DPA alone	40 patients with a mean age of 10 ± 8 month	In comparison to the DPA group, the increases in SNA and ANB angles were noticeably larger in the DPA-FM group. Proclination of upper incisors (U1/NA) and retroclination of lower incisors (L1/NB) were greater in the DPA group than in the DPA-FM group. The increase in ANS-Me length was significantly greater in the DPA-FM group than in the control group.
Buyukcavus M. et al. (2020) [57]	Prospective	55 pz (29 F, 26 M); mean age 11.4 ± 1.06 yo.	Group 1: RME (18 patients); Group 2: Modified Alt-RAMEC (19 patients); Group 3: Skeletal anchorage with FM (18 patients)	ANB angle, dental class, facial profile	Thirty cephalometric measurements were made before and after treatment.	The FM application with skeletal anchorage was the most effective method.
Cevidanes L. et al. (2010) [49]	Consecutive treatment study	BAMP: 21 pz; RME/FM: 34 pz; prepuberal age	BAMP (Bone-Anchored Maxillary Protraction); RME/FM (Rapid Maxillary Expansion with FM)	Cephalometric dentoskeletal changes through Shapiro–Wilk’s test	Changes in cephalometric variables	BAMP protocol significantly improved maxillary advancement, sagittal mandibular changes, vertical control, sagittal intermaxillary ratios, and reduced clockwise mandibular rotation and lower incisor retroclination compared to RME/FM therapy.
Choi Y. et al. (2023) [58]	Retrospective study	50 pz. Grp 1: 25 pz (mean age 9.3 ± 1.1 years); Grp 2: 25 pz (mean age 9.7 ± 1.3 years)	Grp 1: Conventional FM anchored to teeth; Grp 2: Miniplate-anchored FM	Lateral cephalography	FM worn for at least 14 h a day. Cephalometric measurements were analyzed to determine skeletal and dental changes before and after FM treatment.	Miniplate-anchored FM treatment increased protrusion and reduced side effects in Cl III patients.
Fabozzi et al. (2020) [59]	Retrospective observational study	68 patients (38 males and 30 females) with a mean age of 7.5 ± 1.4	SEC III versus RME/FM	SEC III against RME/FM, SNA +1.2 and +1.4 degrees, SNB −1.3 and +1.4 degrees, ANB +2.6 and +2.9 degrees, WITS +3.7 and +2.6 mm	25 patients (10 males, 15 females) with the SEC III protocol were evaluated at the beginning (T1) (mean age 7.5 ± 1.4 years) and at the end of treatment (T2), compared with 32 patients (16 males and 16 females) RME/FM and with (CG) consisting of 23 subjects (12 males, 11 females) untreated.	In comparison to the SEC III group (+1.8 degrees) and the CG (+2.0 degrees), the intermaxillary divergence increased much more in the RME/FM group.
Fisher B. et al. (2018) [44]	Comparative study	34 patients (18 F, 16 M); Alt-RAMEC/FM group: 17 pz; RME/FM group: 17 pz; prepuberal age	Alt-RAMEC/FM and RME/FM	Dentoskeletal changes	Pre-treatment assessment with CBCT; Treatment with Alt-RAMEC/FM or RME/FM; Post-treatment CBCT follow-up	Similar compliance in both groups. No statistically significant differences.
Galeotti et al. (2021) [60]	Randomized controlled trial	42 patients (21 in each group	PS3 versus RME/FM	SNA = 0.4°; *p* = 0.547), SNPg (−1.6°; *p* < 0.001, ANPg (1.4°; *p* = 0.018 RME/FE group vs. PS	42 patients aged 4 to 10 years, Caucasian origin	It improved similarly in both groups (SNA = 0.4°; *p* = 0.54). Decreased SNPg angle (−1.6°; *p* < 0.001) and an increase in ANPg angle (1.4°; *p* = 0.018) were found in the RME/FM group compared with the PS3 group. When compared to the PS3 group, the CoGoMe angle was lower in the RME/FM group (−1.7°; *p* = 0.042). According to regression analysis, there is a correlation between the SN/MP angle at T0 and the differences between T1 and T0 of SNPg and the SN/MP (B = 0.13; *p* = 0.005).
Gencer et al. (2015) [61]	Comparative study	45 children: first group, 15 patients (7 girls and 8 boys; mean age = 11 years); second group, 15 patients (mean age 10 years 9 months); third group, 15 patients (mean age = 10 years 5 months) used as controls.	Double-plate appliance/FM (DPA-FM) combined therapy and FM (FM) therapy	SN (mm) SNA (dg) SNB (dg) ANB (dg) SN/Go-Gn (dg) ANS-PNS/Go-Me (dg) SN/ANS-PNS (dg) ANS-Me (mm) Overbite (mm) Overjet (mm) Molar relation (mm) U6/ANS-PNS (dg) L6/Go-Me (dg) U1/NA (dg) L1/NB (dg)	Lateral cephalometric radiographs of 45 children with skeletal and dental CIIIM (15 treated with DPA-FM, 15 with FM therapy, and 15 as a control group). The paired *t*-test was used to evaluate changes during the treatment. The Duncan test and variance analysis were used to assess group differences.	The SNA and ANB angles, as well as lower facial height, increased dramatically with the DPA-FM and FM appliances. Both appliances worked well. The considerable sagittal changes in the FM group’s lower incisors and pogonion could be attributed to the limitation impact of acrylic blocks in the DPA-FM group.
Giunti et al. (2021) [62]	Clinical study	29 patients (13 females and 16 males) with RME/FM and 23 patients (13 females and 10 males) with prepubertal RMR	RME/FM versus RMR	Greater (SNA +1.5 mm, *p* = 0.031) and significantly greater improvements in ANB and Wits assessment (+1.9 degrees, *p* = 0.002 and +2.2 mm, *p* = 0.012, respectively) were recorded in the RME/FM group.	2 prepubertal patients. The mean age for the RME/FM group was 7.4 ± 1.6 years at T1, 9.0 ± 1.7 years at T2, and 15.1 ± 1.4 years at T3. The mean age for the RMR group was 7.7 ± 2.5 years at T1, 9.4 ± 2.3 years at T2, and 14.8 ± 1.3 years at T3. Mandibular functional or as a retention device after RME/FM.	The goal is to produce favorable correction of SCIIIM imbalance; the RME/FM protocol should be preferred over RMR. RMR may be useful in the treatment of pseudo Cl III. The RME/FM group displayed SNA +2.3 degrees, SNB −1.3 degrees, and ANB and Wits displayed +3.6 degrees and +2.6 mm, respectively, compared to the RMR Group.
Godt et al. (2008) [30]	Comparative study	41 patients (17 males, and 14 females); mean age 6.98 (FM) and 7.12 (removable appliances)	Removable appliances with or without face masks	SNA, SNB, ANB, Wits (mm), SN-Pog, SN-MeGo, *y*-axis, Go2 (NGoMe), NS-SpP, mandibular angle, length of the maxilla (mm), length of the mandible (mm), angulation of upper first incisors, angulation of lower first incisors, overjet (mm), overbite (mm)	At baseline and at the conclusion of early therapy, casts were collected, and lateral cephalograms were analyzed. Two different treatment plans were contrasted. Only detachable functional orthopedic appliances were used in the first group (FOA group), whereas removable appliances and face masks placed on a cemented maxillary expansion appliance were used in the second group (face mask group).	Positive changes in overjet and Wits values were observed in both groups. Furthermore, a change in mean ANB values (+0.9°) was achieved in the face mask group. Mandibular angles were reduced in the FOA group. The face mask group exhibited dorsal mandibular rotation with lower SNB values (0.8°). Early treatment of prognathism is an important choice.
Husson et al. (2016) [63]	Randomized controlled trial	Thirty-two patients (8 males and 8 females); mean age 7.98 ± 0.68 years	The modified tandem appliance (MTA) vs. the FM (FM) with rapid maxillary expansion	SNA (dg), ANB (dg), CoA (mm), CoGn (mm), N⊥FH-A (mm), N⊥FH-Pog (mm), SN/SPP (dg), SN/GoMe (dg), Bjork’s sum (dg), PointA-TV (mm), PointA-TW (mm), Pog-TV (mm), Pog-TW (mm), Uppermolar-TV (mm), Uppermolar-TW (mm), Upperincisor-TV (mm), Upperincisor-TW (mm), Lowermolar-TV (mm), Lowermolar-TW (mm), Lowerincisor-TV (mm), Lowerincisor-TW (mm), Overjet (mm), Overbite (mm)	The sample was divided into two equal groups to be treated with either MTA or FM. Lateral cephalometric radiographs were obtained before treatment and after a 2 mm positive overjet was achieved. Intragroup comparisons were performed using a paired-sample *t*-test, and intergroup comparisons were performed using a two-sample *t*-test at the *p* ≤ 0.05 level.	Both appliances showed similar effects (similar significant increase in the SNA and ANB angles) apart from less clockwise rotation of the mandible, less retrusion of the lower incisors, and greater uprighting of the lower molars in the MTA group.
Kilicoglu et al. (2017) [64]	Clinical study	6 patients treated with Jasper Jumper, 17 with FM, and 13 CG; mean age 10 years	Jasper Jumper versus FM Delaire	The upper incisors (is/OLp), molar teeth (ms/OLp), and maxillary base (ss/OLp) all shifted substantially forward in both treatment groups. In the modified Jasper Jumper group, the anterior crossbite was improved by upper incisor protrusion (is/OLp-ss/OLp), lower incisor retrusion (ii/OLp-Pg/OLp), and increased overjet (is/OLp-ii/OLp), whereas in the face mask group, it was improved by lower incisor retrusion (ii/OLp-Pg/OLp) and increased overjet	Cephalometric assessment was used with Pancherz analysis. CIII improvements in both groups are generated with the forward movement of the upper first molar and backward movement of the lower first molar	The Jasper Jumper group overjet correction of 4.63 mm was due to 51.4% skeletal and 48.6% dental changes; the CIII molar correction of 4.77 mm was due to 49.9% skeletal and 50.1% dental changes. In the Delaire facial mask group, the overjet correction of 5.17 mm was due to 70.6% skeletal and 29.4% dental changes, and the CIII molar correction of 4.87 mm was due to 75% skeletal and 25% dental changes.
Lee et al. (2023) [65]	Retrospective study	40 subjects (26 females and 14 males); mean age 7.7 years	Protraction FMs with forehead straps (PFFS) or Petit type FMs (PTF)	SNA (°), SNB (°), A point, N Perp (mm), Pog-N Perp (mm), ANB difference (°), Wits appraisal (mm), Maxillary length (mm), Mandibular length (mm) Articular angle (°), FMA (°), PFH/AFH (mm), Palatal plane angle (°), Facial angle (downs) (°), U1 to FH (°), IMPA (°) Incisor overbite (mm), Incisor overjet (mm), Upper lip EL (mm), Lower lip EL (mm)	Patients with Cl III were treated with protraction FMs with forehead straps (PFFS) or Petit type FMs (PTF).	Both PFFS and PTF showed no significant differences in most skeletal and dental changes, except for overbite.
Liu et al. (2020) [66]	Comparative study	28 subjects (16 girls and 12 boys); mean age 11 years 1 month (FM with expansion group), mean age 11 years 6 months (FM only group)	Banded versus modified appliances	Co-A; Co-Gn; maxillary depth (NA/FH); facial depth (NPg/FH); SNA; SNB angle; ANB angle; Wits appraisal; maxillary height (NCF/CF-A); palatal plane (ANSPNS/FH); facial axis (N-Ba/CC-Gn); SN/GoGn; GnGoAr. SN/occlusal plane; molar relationship; overjet; overbite; U6PTV; U1-NA (mm); U1-NA (°); NSBa; SN. 1, ANSx; 2, Pgx; 3, ANSy; 4, Pgy. U6x; 2, U1x; 3, U6y; 4, U1y.	20 patients each got maxillary protraction using a banded appliance and a modified device. All individuals had their cephalometric radiographs taken before and after the procedure, which were examined. For statistical analysis, the Wilcoxon ranks test and the paired *t*-test were utilized.	Compared to patients in the banded appliance group, patients in the modified appliance group required less time for tissue treatment. The banded appliance group, however, demonstrated a greater increase in mandibular plane angle, anterior facial height, total facial height, mesialization of maxillary molars, and proclination of maxillary incisors (*p* 0.05) as compared to the modified appliance group. The newly developed modified appliance may be a successful technique for treating increasing Cl III patients with maxillary deficit since it might reduce treatment times, increase treatment efficacy, and diminish anchoring loss.
Liu et al. (2021) [67]	Clinical study	9 patients: FM group (7 males and 5 females); mean age 9.53 ± 1.37 years; rapid maxillary expansion (RME/FM) group (6 males and 6 females)	3 groups compared FM, RME/FM, Alt-RAMEC/FM.	Alt-RAMEC showed statistically more significant maxillary advancement than the other groups (A-VRP, 3.87 mm vs. 3.04 mm [RME/FM], vs. 2.04 mm [FM]); *p* < 0.05. There were more skeletal effects.	In the Alt-RAMEC/FM technique, the skeletal effects were more prevalent (88.7%) during the overjet correction.	In the treatment of prepubescent individuals with maxillary deficiency, the modified Alt-RAMEC protocol combined with FM showed more beneficial skeletal outcomes than FM and RME/FM protocols.
Maspero C. (2012) [22]	Retrospective	104 pz (53 F, 51 M); age 5–9 yo.	Grp 1: TSME; Grp 2: RME/FM	Lateral cephalography	104 patients with Cl III skeletal relationship; 52 were treated with TSME (Grp 1) and 52 with RME/FM (Grp 2). Cephalograms taken before treatment (T0) and after retention (T1).	RME/FM led to the significant forward movement of the maxilla.
Minase et al. (2019) [35]	Prospective clinical trial	39 patients; age 6–12 yo.	Grp 1: Reverse twin block with lip pads-RME (RTBLP-RME); Grp 2: FM with RME (FM-RME) Grp 3: Control	Lateral cephalography	39 patients with Cl III divided into 3 groups: RTBLP-RME, FM-RME, and Control. Treatment duration: 9 months. Cephalograms taken at T1 (beginning) and T2 (after 9 months) for both groups.	RTBLP-RME showed more significant sagittal changes, non-significant increases in vertical measurements, and greater impact on maxillary advancement and posterior positioning of the mandible with minimal dental compensation compared to FM-RME.
Ngan P. et al. (2015) [18]	Retrospective study	20 pz (8 M, 12 F); mean age 9.8 ± 1.6 yo.	RPE on teeth with facial mask and RPE Hybrid Hyrax anchored on bone with FM	Skeletal and dentoalveolar changes	Cephalometric analysis based on measurements from Bjork and Pancherz, McNamara, Tweed, and Steiner analyses	The teeth-anchored group showed increased proclination of maxillary incisors, improved overjet correction, and molar relationship correction.
Parayaruthottam P. et al. (2018) [68]	Retrospective study	18 pz (10 F, 8 M); mean age 10.1 yo.	Alt-RAMEC/protrusion or RME/protrusion	Skeletal, dental, and soft tissue parameters	Cephalograms of two groups (Alt-RAMEC and RME) were analyzed pre- and post-treatment	Group 2 (Alt-RAMEC) showed significant forward movement of the maxilla, backward and downward rotation of the mandible, and proclination of maxillary incisors compared to Group 1.
Pavoni et al. (2009) [69]	Comparative study	9 FM/BB patients included 22 subjects (12 girls and 10 boys). RME/FM included 17 subjects (10 girls and 7 boys).	FM/BB versus RME/FM	The only exceptions were the sagittal maxillary angular and mandibular measurements (SNA°, SNB°), which were significantly larger in the FM/BB group and the position of upper and lower incisors that were significantly more prominent in the FM/BB group.	There were 22 subjects in FM/BB, including 12 girls and 10 boys. The FM/BB group had a mean age of 8.7 +/− 1.2 years before treatment (T1), 10.4 +/− 1.3 years after active therapy (T2), and a mean treatment duration of 1.7 +/− 0.8 years. Ten girls and seven boys were among the 17 individuals in the RME/FM sample. At T1 and T2, the average age was 7.8 +/− 1.8 and 9.3 +/− 1.9, respectively. The average observation period was 1.5 +/− 0.6 years. At T1 and T2, lateral cephalograms were examined. A *t*-test using an independent sample was used to evaluate changes from T2 to T1 in the two groups (*p* 0.05).	No differences were shown in the duration treatments for any measurements, both sagittally and vertically in T1 and T2
Perillo et al. (2016) [70]	Retrospective observational study	68 patients (38 males and 30 females) with mean ages of 7.5 ± 1.4 (T1) and 8.7 ± 1.4 (T2)	SEC III versus RME/FM	SNA +1.2 and +1.4 degrees, SNB −1.3 and −1.4 degrees, ANB +2.6 and +2.9 degrees, and Wits +3.7 and +2.6 mm obtained with both treatments; SEC III versus RME/FM	25 patients (10 males, 15 females) with the SEC III protocol were evaluated at the beginning (T1), mean age (7.5 ± 1.4 years), and at the end of treatment (T2), compared with 32 patients (16 males and 16 females) for RME/FM, and with the CG consisting of 23 subjects (12 males, 11 females) untreated.	The RME/FM group has a greater 31 intermaxillary divergences than the SEC III group (+1.8 degrees) and CG (+2.0 degrees).
Seehra et al. (2012) [51]	Retrospective comparative study	31 PFM (*n* = 9) or RTB (*n* = 13) patients and matched untreated controls (*n* = 10)	PFM versus RTB	SNA, SNB, and ANB; *p* < 0.001	12 patients treated and 10 untreated	Dentoalveolar effects RTB therapy better than PFM (*p* < 0.001) and retro-inclination of the lower incisor *p* < 0.001
Seiryu et al. (2020) [71]	Single-center, prospective randomized controlled trial	39 patients (24 males and 15 females); FM group mean age 10 years 5 months ± 1 year 8 months; (FM group) 11 years 1 month ± 1 year 3 months (FM + MS group)	FM and FM in combination with a mini-screw	SNA, SNB, ANB, SN-ANS, N-Me, U1-SN, PTM-U6/NF, U6/NF, L6/MP, MP-SN, Facial A, Y-axis	Patients were divided into two groups at random. Patients in one group received FM therapy (FM group), whereas those in the other group received FM therapy in addition to having a mini-screw inserted into the palate and secured to the lingual arch.	SNA, SN-ANS, and ANB values were significantly increased in the FM + MS group rather than the FM group, so FM + MS therapy delivers orthopedic forces more efficiently. Proclamation of maxillary incisors increased more significantly in the FM group, with more negative side effects compared to the FM + MS group.
Tortop et al. (2007) [72]	Comparative study	42 children (23 girls and 19 boys); mean age 11 years 1 month (FM plus expansion); mean age 11 years 6 months (FM group); mean age 10 years 4 months (control group)	FM with or without expansion	CoA, CoGn (mm), Maxillomandibular differential (mm), Maxillary depth (°), Facial depth (°), SNA angle (°) SNB angle (°), ANB angle (°), Wits appraisal (mm), Maxillary height (°), Palatal plane to Frankfort horizontal (°), Facial axis (°), SNGoGn (°), GnGoAr (°), Occlusal plane (°), Molar relationship (mm), Overjet (mm), Overbite (mm), U6PTV (mm), U1-NA (mm), U1-NA (°), NSBa (°), SN (mm)	Pre-treatment and posttreatment lateral cephalograms were used. They were divided into the group with the FM with expansion group (FMEXP), the group with the FM only (FM), and a control group.	In both treatment groups, the maxilla’s forward displacement was noticeably higher than in the control group. The FM group’s rise in maxillary molar extrusion differed considerably from that of the control group. In comparison to the control group, the FMEXP group exhibits a large rise in the mandibular plane angle and a considerable reduction in the face axis. The FM group saw a greater increase in molar connection than the FMEXP group. FM therapy, which provides dental and skeletal accomplishments, can be effective for CIII treatment.
Tortop et al. (2018) [73]	Comparative study	76 patients (32 females and 44 males); mean age 10 years	Three groups treated with MTTBA, FM, and CG	Decreases in SNA and ANB and decreases in SNB in both treatment groups compared with the control group (*p* < 0.001); change in SN/GoGn in treatment subjects. Branch width (DC) (*p* < 0.01) and mandibular posterior space (CLMD) (*p* < 0.001) increased in all groups.	The evaluation was performed on cephalometric radiographs at time T1, before treatment and at T2, after obtaining an overjet of 2–3 mm.	Cephalometric changes in T1 to T2 groups; SNA and ANB increased in the MTTBA group (*p* < 0.01); overbite decreased. The overjet and molar relationship increased significantly (*p* < 0, 001). Branch width (DC); CPg (*p* < 0.01); posterior mandibular space (CLMD) (*p* < 0.001). In the FM group, SNA and ANB values increased but with a decrease in SNB.
Vaughn et al. (2005) [74]	Controlled randomized clinical trial	46 patients (22 females and 24 males); mean age 7.3833 (group with FM with palatal expansion), 8.1086 (group with FM without palatal expansion)	FM with or without expansion	Maxilla anteroposterior, SNA Maxillary depth, ANS A-point Nasion perpendicular to A-point, Maxillary length, Maxilla vertical PNS, ANS A-point SN-palatal plane, Mandible anteroposterior, SNB, Facial depth, Nasion perpendicular to pogonion, Mandibular length, B-point Pogonion, Mandible vertical, GO-GN-SN, Lower facial height, B-point, Pogonion, Maxilla/mandible anterior/posterior, ANB angle, Mx/Mn difference, Wits, Convexity	Randomly chosen groups of patients were given the FM with palatal expansion, the FM without palatal expansion, or they were the control group. An x–y coordinate system, an occlusal-plane analysis, and classic cephalometric data were all used in the cephalometric study.	No significant differences (*p* 0.05) were found between the expansion and non-expansion groups for any measurable variable according to Student’s *t*-tests. Significant changes from the control group show that early FM therapy, whether it includes or excludes palatal extension, is successful in correcting skeletal Cl III.
Wendl et al. (2017) [75]	Retrospective study	61 patients (41 males and 20 females); mean age 7.8 ± 1.7 years at T0	Chin cup or FM	SNA; Cond.-A (mm); SNB; Cond.-Gn (mm); ANB; MM diff. (mm); Ar-Go-Me; Ar-Go (mm); NSBa; Go-Me (mm); ML-NSL; Spp-Spa (mm); NL-NSL; UCI/SN; ML-NL; LCI/ML; Bjork’s sum; UL-EL (mm); Wits (mm); LL-EL (mm); PFH:AFH (ratio)	Two examiners independently analyzed data from cephalograms, casts, and orthopantomograms of patients with Cl III syndrome in a pre-treatment situation (T0), post-treatment situation (FM or chin cup) after correction of the malocclusion (T1), and long-term follow up situation 15–20 years later (T2).	For the therapy of Cl III, either an FM or chin cup is beneficial. Because of excessive mandibular growth or a lack of maxillary catch-up growth and impairment of the maxillomandibular connection, the subgroup receiving chin cup treatment had worse long-term stability.
Yagci and Uysal (2010) [37]	Randomized control study	69 patients (33 males and 36 females); mean age 9.2 ± 1.4 years (conventional FM treatment group); mean age 9.3 ± 1.6 years (modified FM treatment group); mean age 9.8 ± 1.9 years (control group)	Conventional and modified FM therapies with rapid maxillary expansion	SNA, SNB, ANB, SN-MP, SN-PP, A to N perp, Pg-Na perp, N-Me, N-ANS, ANS-Me, interincisal angle, U1-NA, U1-PP, l1-NB, L1-MP, nasolabial angle, upper lip to E plane, lower lip to E plane	The sample was split into three groups: the traditional FM group (Group 1), the modified FM treatment group (Group 2), and the control group (Group 3), each of which included 24 patients. The Kruskal–Wallis test was used to study intergroup changes, while the non-parametric Wilcoxon’s test was used to assess intra-group comparisons. The Mann–Whitney test for independent samples and Bonferroni’s correction were used to further evaluate the statistical significance of intergroup differences (*p* = 0.016).	SNB alterations in group 1 were lower than in the control group. Increases were seen in SNA, ANB, SN-MP, A to N perp, and upper lip to E plane. SNB, U1-NA (mm), U1-NA (°), and Pog to N perp (mm) increases were lower in group 2 compared to the control. SNA, ANB, SN-MP, A to N perp, and Upper lip to E plane all showed increases. Patients with a retrognathic maxilla in Cl III can benefit from the modified FM appliance. The maxilla advanced anteriorly and translated without rotating as a result of FM treatments with expansion, while the mandible progressed rearward and downward in both treatment groups.
Yavan et al. (2023) [76]	Randomized controlled trial	45 subjects with mild Cl III (20 females and 25 males); mean age 10.54 years (FM/RPE group); mean age 10.49 years (RF group); mean age 10.66 years (control group)	Reverse forsus (RF) and FM/rapid palatal expansion (FM/RPE)	ANB, overjet, sagittal lip relationships, anterior and inferior traction of the maxilla, as well as proclinating mandibular and maxillary incisors	45 participants with mild CIIIM had their lateral cephalograms taken both before and after treatment. A group was given an FM/RPE appliance, a different group was given an RF appliance, and a control group went untreated. One-way analysis of variance, the Kolmogorov–Smirnov test, the Kruskal–Wallis test, the paired-samples *t*-test, and the Wilcoxon test were used for the statistical analyses, with a *p* value of 0.05 being considered statistically significant.	Intermaxillary and interdental improvements resulted from both procedures. When compared to FM/RPE therapy, the RF appliance mostly exhibited dentoalveolar effects and had little effect on the maxilla.

## Abbreviations

Alt-RAMEC	Alternate maxillary expansion and constriction
ANB	Point A–Nasion–Point B
BAMP	Bone anchored maxillary protraction
CBCT	Cone beam computed tomography
CIII	Class III
CIIIM	Class III malocclusion
Co	Condyle
DPA	Double-plate appliance
FM	Facemask
FM/BB	Bite block and face mask
Gn	Gnation
Go	Gonion
Me	Menton
MTTBA	Modified tandem traction bow appliance
N	Nasion
PFM	Proctation face mask
PNS	Posterior nasal spine
PS3	Pushing splits 3
RME	Rapid maxillary expansion
RME/FM	Rapid maxillary expansion and facial mask
RMR	Removal mandibular retractor
RTB	Reverse twin block
RTBLP-RME	Modified reverse twin block with rapid palate expansion
S	Saddle
Cl III	Skeletal class III malocclusion
SEC	Splints, class III elastics, and chin cup
Sna	Anterior nasal spine
SNA	Saddle–Nasion–Point A
SNB	Saddle–Nasion–Point B
TSME	Transverse sagittal maxillary expander
